# Supplementary material for: Surface Microbiome Profiling of Dental Elevators Using Third‐Generation Sequencing: Implications for Infection Control in Dental Practice
Source: Microbiologyopen. 2025 Dec 8;14(6):e70178. doi: 10.1002/mbo3.70178 (PMC12685768; doi:10.1002/mbo3.70178)
Supplement: Supplementary file 1 — customer_backup. [file MBO3-14-e70178-s001.zip › customer_backup/customer_backup/function_analysis/BugBase/treat/otu_contributions/genus/treat.genus.Forms_Biofilms.pdf]

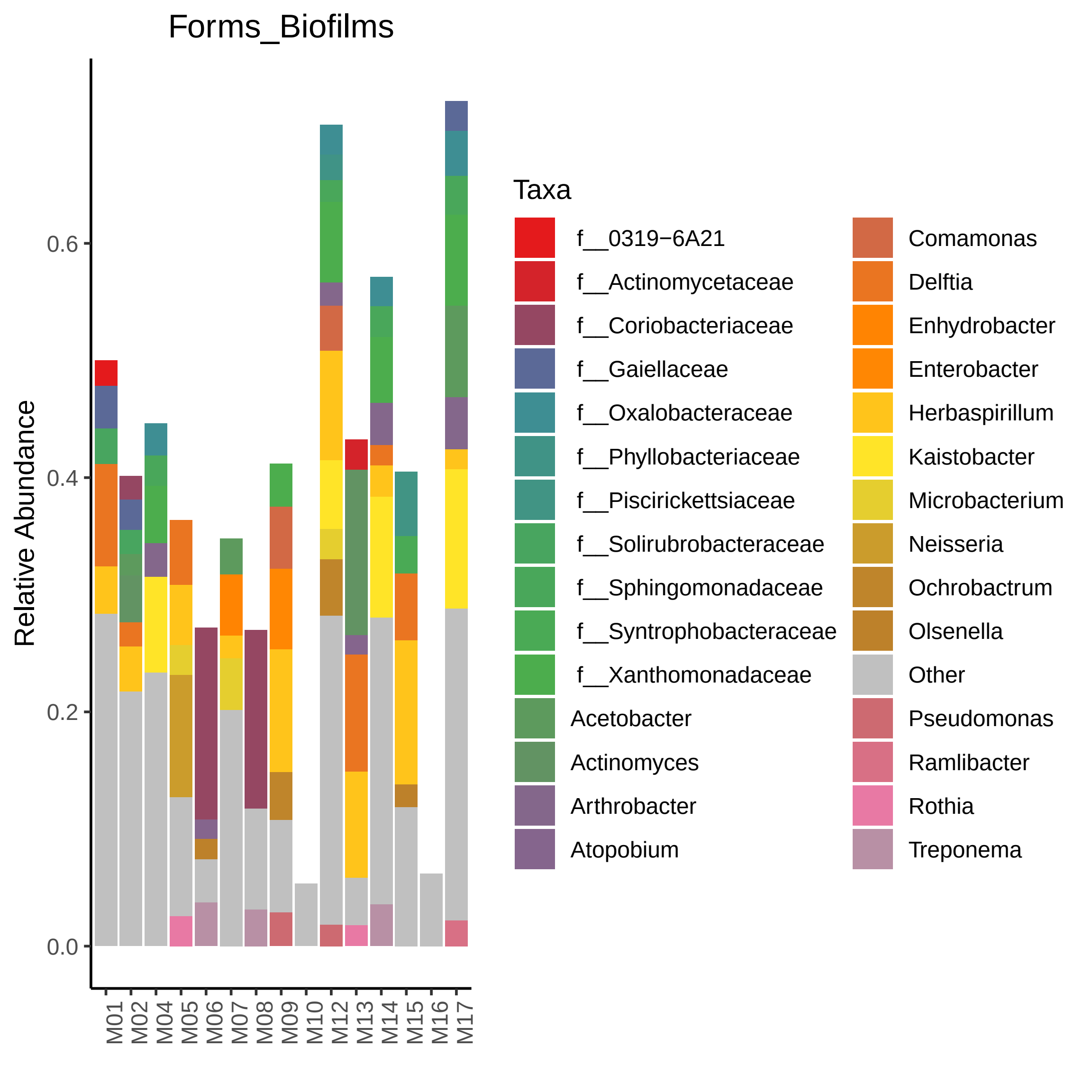

Forms_Biofilms
Relative Abundance
0.0
0.2
0.4
0.6
M01
M02
M04
M05
M06
M07
M08
M09
M10
M12
M13
M14
M15
M16
M17
Taxa
f__0319−6A21
f__Actinomycetaceae
f__Coriobacteriaceae
f__Gaiellaceae
f__Oxalobacteraceae
f__Phyllobacteriaceae
f__Piscirickettsiaceae
f__Solirubrobacteraceae
f__Sphingomonadaceae
f__Syntrophobacteraceae
f__Xanthomonadaceae
Acetobacter
Actinomyces
Arthrobacter
Atopobium
Comamonas
Delftia
Enhydrobacter
Enterobacter
Herbaspirillum
Kaistobacter
Microbacterium
Neisseria
Ochrobactrum
Olsenella
Other
Pseudomonas
Ramlibacter
Rothia
Treponema
